# The Influence of Orthodontic Bracket Base Design and Bonding System on Shear Bond Strength

**DOI:** 10.3390/jfb17030110

**Published:** 2026-02-24

**Authors:** Maria Manuela Nardin, Alin Gabriel Ionescu, Alexandra Elena Done, Cosmin Mihai Mirițoiu, Paula Adriana Pădeanu, Anne Marie Rauten, Luminița Dăguci, Cristina Teodora Preoteasa, Veronica Mercuț

**Affiliations:** 1 Department of Prosthetic Dentistry, University of Medicine and Pharmacy of Craiova, 200349 Craiova, Romania; nardin.mmanuela@gmail.com (M.M.N.); luminita.daguci@umfcv.ro (L.D.); veronica.mercut@umfcv.ro (V.M.); 2 Department of History of Medicine, University of Medicine and Pharmacy of Craiova, 200349 Craiova, Romania; 3 Department of Scientific Research Methods-Ergonomics, Faculty of Dentistry, “Carol Davila” University of Medicine and Pharmacy, 020021 Bucharest, Romania; donedalexandra@yahoo.com; 4 Department of Applied Mechanics and Civil Constructions, Faculty of Mechanics, University of Craiova, 200585 Craiova, Romania; cosmin.miritoiu@edu.ucv.ro (C.M.M.); padeanuadriana2000@yahoo.com (P.A.P.); 5 Department of Orthodontics, University of Medicine and Pharmacy of Craiova, 200349 Craiova, Romania; annemarie.rauten@umfcv.ro

**Keywords:** shear bond strength, bracket, self-etching, anchor pylons, 80-gauge mesh

## Abstract

The success of orthodontic therapy depends on the effective, continuous application of forces to teeth. Therefore, an essential element of the treatment is the adhesion between the bracket and enamel. The purpose of this study was to evaluate the influence of bracket base design and bonding system on shear bond strength. The study was conducted on eighty extracted premolars which were randomly divided into four groups of twenty teeth each, using two types of metal brackets (80-gauge mesh and anchor pylons base design) and two types of bonding systems (conventional and self-etching). The combination of bracket and bonding system resulted in four distinct configurations of bracket bonding, with each configuration tested on twenty teeth. Shear bond strength testing was performed using a Laryee Universal Testing Machine. The obtained values were statistically analyzed. Slightly higher shear bond strength values were recorded for brackets with anchor pylons bonded using the conventional bonding system (13.32 ± 4.20 N/mm^2^), whereas the lowest values were recorded for the same bracket base design bonded with the self-etching system (11.10 ± 4.50 N/mm^2^). Nevertheless, ANOVA test did not reveal statistically significant differences between the two bracket types or between the two bonding techniques in terms of shear bond strength and force values and no significant interaction effects were observed. Considering the obtained results, several additional factors must be taken into account when evaluating the shear bond strength of orthodontic brackets.

## 1. Introduction

Currently, fixed appliances represent the gold standard in orthodontic treatment due to their ability to achieve optimal tooth movement [1]. Orthodontic brackets represent an essential component of fixed appliances. Attached to the vestibular or lingual surface of the tooth, they transmit the forces generated by the archwires or other accessories, thereby causing tooth movement [2].

In the early days of orthodontic treatment with fixed appliances, brackets were welded to stainless steel or gold bands. Applying them was a laborious process that was uncomfortable for the patient [3].

Adhesion concepts and techniques are constantly evolving, the concept of „generation” being used to emphasize each significant improvement. Each generation aimed to reduce the number of components involved in treatment, minimize procedural steps, achieve a faster technique and improve the chemical formula for a stronger adhesive bond [4].

The advent of bioactive and biomimetic adhesion systems represents a paradigm shift from passive mechanical retention to active therapeutic integration.

The evolution of biomimetic materials marks a critical point in the development of dental adhesives, shifting the focus toward the replication of the structure and function of tooth tissues [5,6]. These materials are engineered to reproduce the mechanical, chemical, and biological properties of enamel and dentin [7,8].

Bioactive dental adhesives interact with biological tissues to provide therapeutic advantages. Among these, Giomers stand out as “intelligent” hybrid materials which combine the mechanical durability and aesthetic quality of resin composites with the protective benefits of glass ionomers. They incorporate Surface Pre-Reacted Glass-ionomer (S-PRG) fillers, which facilitate the release and recharge of fluoride, calcium, and phosphate ions. This ion exchange provides buffering capacity against acidic challenges and promotes remineralization at the interface between bracket and tooth [9,10].

In orthodontics, the materials involved in the adhesion process must have certain properties, given their use in the oral cavity: biocompatibility, resistance to solubility and to the physical and chemical attack of the oral environment. Also, they must achieve an optimal bond to the tooth surface, provide sufficient working time, immediate sealing to prevent microleakage, easy removal of excess material and easy handling [3,11].

The adhesive bond between bracket and tooth should be strong enough to withstand the forces generated by the dento-maxillary apparatus and the forces induced during the orthodontic treatment. Furthermore, when the brackets are removed from the tooth surface, either accidentally or at the end of treatment, there should be no risk of damage to the enamel.

An optimal adhesive bond between the bracket and the tooth reduces the frequency of accidental bracket failure, while also reducing the risk of prolonging treatment time and financial costs [12,13,14].

Bracket bond failure can be determined by patient-related factors (age, gender, oral hygiene, and dietary preferences) [15,16], the type of malocclusion [13,17], the location of the bracket (tooth number, maxillary or mandibular arch, anterior or posterior region) [17,18], but also by procedural factors (difficulties in ensuring proper isolation during bracket bonding, improper adaptation of the bracket to the tooth surface, type of etching and adhesive material, type of bracket, size and bracket base design) [11,19,20,21].

Stainless steel metal brackets are the most used in orthodontic practice [22]. The metal base does not react chemically with the adhesive resin. Therefore, the adhesive bond between the bracket and the tooth is based on the micromechanical retention provided by the design of the bracket base [23,24,25]. The most used brackets have a mesh base. The mesh can be characterized by different variables: mesh size, the number of mesh layers and the diameters of the mesh wires [2]. Brackets incorporating a mesh base are manufactured as “two piece” brackets, having the wings brazed to the metal base, while the retention mesh is attached on the outer surface of the base [26]. There are also “one piece” brackets, where the physical retention of the adhesive is enhanced by cutting grooves or pillars into the base of the bracket. This results in irregular areas where the adhesive will penetrate [26]. Sandblasting, micro-etching and laser-etching of the bases can improve the strength of the adhesive bond by increasing the contact surface [27].

The conventional bonding systems or the three step bonding technique with separate enamel etching is the gold standard for achieving an optimal adhesive bond [28]. Strong or prolonged enamel etching can cause damage to the enamel around the bracket and its cracking during the debonding process. Furthermore, the technique involves multiple clinical steps, so the likelihood of errors is increased [29]. Conventional adhesion is achieved through the creation of a high-energy substrate using phosphoric acid demineralization. This process generates a microporous enamel topography for the infiltration of amphiphilic monomers [30]. The resulting interfacial structure–property relationship is defined by the formation of “resin tags”, whose efficacy in providing micromechanical interlocking is dependent on the monomer’s capacity to displace residual moisture and minimize polymerization shrinkage [31,32]. However, the hydrolytic stability of this interface remains a critical vulnerability; wetting or salivary contamination facilitates incomplete resin infiltration, creating zones that serve as precursors for bond failure and the development of white spot lesions [33].

Self-etching bonding systems were developed to significantly reduce chair time and the risk of enamel surface contamination during the bracket bonding process. The application of demineralizing acid and primer is performed in a single step [34,35,36].

The existing studies conclude that self-etching systems are gentler than systems that use separate etching with 37% phosphoric acid [37].

Shear bond strength of self-etching systems is a controversial topic, as there are studies in the literature that report similar values [38], while others report lower values compared to conventional systems [39].

Shear bond strength testing is one of the essential factors for evaluating the performance of adhesive materials [40]. This test measures the maximum stress that a material can withstand before failure, focusing primarily on the interface between the adhesive and the substrate, under the action of a shear force [34].

Although shear bond strength of orthodontic brackets has been widely studied, many published studies provide limited information regarding bracket base design, which can significantly influence bonding outcomes. While 80-gauge mesh bases achieve retention primarily through adhesive penetration into the mesh structure, anchor pylon base designs rely on discrete retentive projections that interact differently with orthodontic adhesives.

Conventional metal brackets with mesh bases, particularly those with 80-gauge mesh, have been evaluated in shear bond strength studies [41,42,43]. While anchor pylon brackets are available for clinical use, their shear bond strength has not been extensively evaluated. To our knowledge, the literature regarding the bonding efficacy of this specific base design remains limited. Addressing this research gap is essential to inform clinicians about optimal bracket selection, highlighting the need for further investigation.

The aim of this study was to compare the shear bond strength of various combinations of brackets with different base designs and adhesive systems from different generations, in order to identify the most effective combination. For this purpose, two types of brackets were selected: metal brackets with an 80-gauge mesh base and metal brackets with anchor pylons. Regarding the adhesive systems, in this study conventional and self-etching systems were used.

## 2. Materials and Methods

### 2.1. Study Design

An experimental, cross-sectional and analytical study was conducted. The shear bond strength was tested for two types of brackets and two types of adhesive systems on 80 teeth, randomly divided into 4 groups of 20 teeth each. Each tooth was tested at a single point in time. All procedures were performed by the same operator.

### 2.2. Study Group

**Figure 1:**
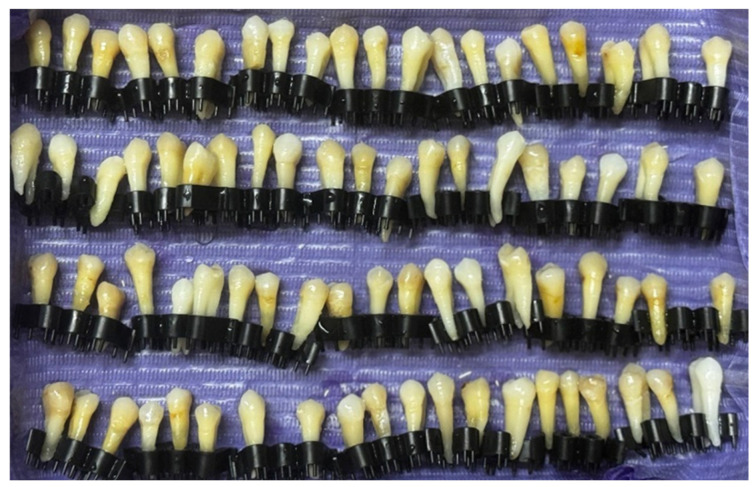
Premolars included in the study.

In this study, 80 premolars extracted for orthodontic purposes were used (Figure 1). These were distributed into 4 different groups, depending on the adhesive system and type of bracket tested. The sample size was computed using G*Power 3.1.9.7 (from Heinrich Heine University Düsseldorf, Germany), considering a significance level α of 0.05, a power 1 − β equal to 0.8, and a medium to large effect size (with an awareness of practical significance), resulting in a study requirement of a minimum of 74 teeth. This sample size was confirmed by reviewing comparable in vitro studies in the literature [44,45,46,47].

### 2.3. Eligibility Criteria

Inclusion criteria: A total of 80 premolars that had been extracted without trauma, with intact vestibular enamel were selected.

Exclusion criteria: Teeth that had been subjected to any chemical pretreatment (e.g., hydrogen peroxide), teeth with cracks caused by the presence of extraction forceps and teeth with caries or developmental defects were excluded.

**Figure 2:**
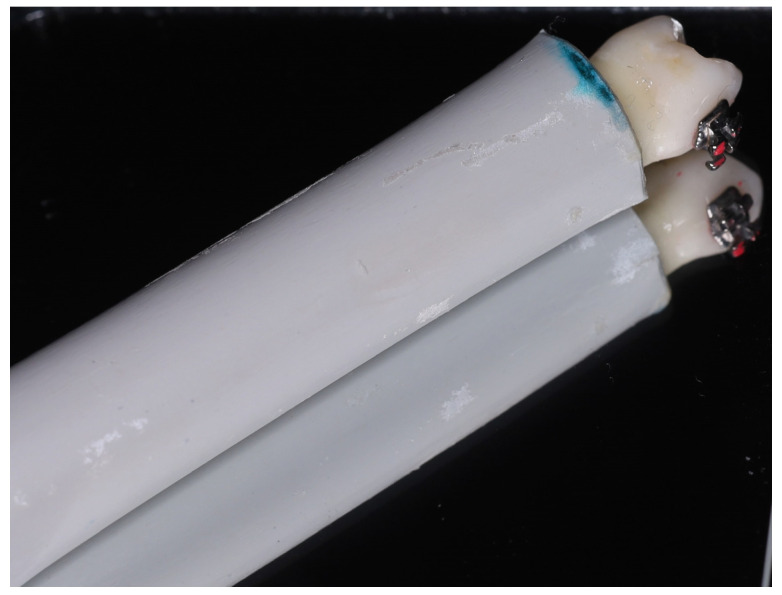
Tooth sample prepared for shear bond strength testing.

The teeth were cleansed of soft tissue and blood debris and were polished with fluoride-free prophylactic paste [48]. Subsequently, until the brackets were applied, the teeth were stored in distilled water, which was changed weekly to prevent bacterial growth. Right after bracket placement, for shear testing, each tooth was fixed in self-curing acrylic resin blocks (Struers GmbH, Willich, Germany) so that the roots were completely embedded up to the cemento-enamel junction. Each tooth was oriented so that the vestibular surface of the crown was parallel to the direction of the force during shear bond strength testing [48]. To avoid damage to the acrylic resin during testing, the resin was poured into rigid PVC tubes (Figure 2).

To better distinguish the groups, the blocks were labeled according to Table 1.

**Figure 3:**
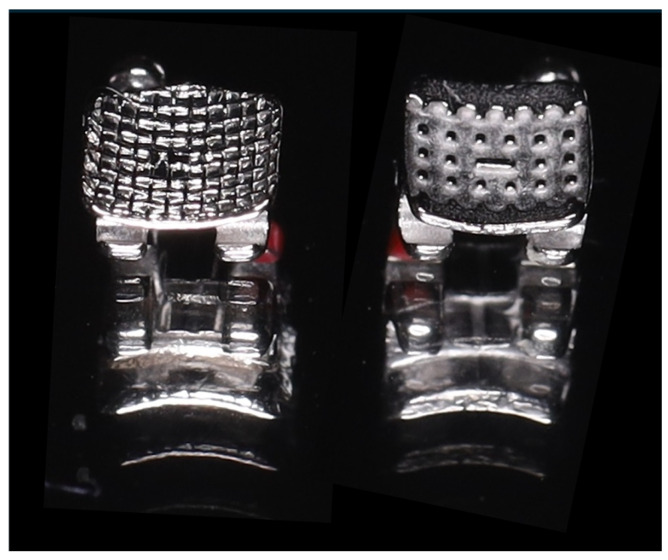
Metal brackets used in the study.

In this study, two types of stainless steel brackets were used for upper premolars (torque −7° and angulation 0°), with a slot size of 0.22″ × 0.28″, MBT prescription (Figure 3). The difference was in the design associated with the bracket base. Therefore, brackets with a surface area of 13.2 mm^2^ with an 80-gauge mesh base and brackets with a surface area of 13.8 mm^2^ with anchor pylons were used.

**Figure 4:**
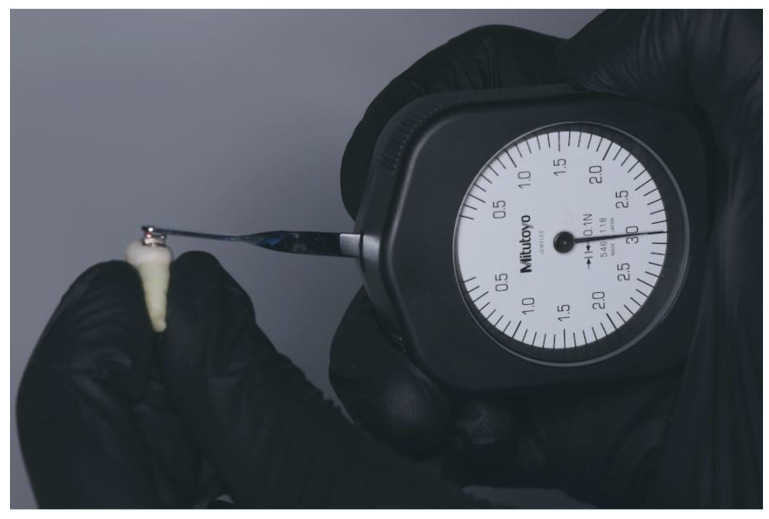
Measurement of compression force for each bonded bracket.

For the conventional bonding technique, the vestibular enamel etching was conducted with 37% phosphoric acid gel for 30 s. The acid was washed off and the vestibular surface was dried until it appeared matte and chalky white. The next step was to apply a thin layer of primer. This was applied with a brush, then blown with a gentle jet of air and light-cured for 3 s. Subsequently, the brackets were bonded on the vestibular surface of the tooth. Each bracket was subjected to a compression force of 300 gF (3 N) with a dynamometer (Mitutoyo, Japan) (Figure 4). The excess resin was removed with a probe. This was followed by photopolymerization of the bracket.

For the self-etching technique, a two-compartment system was used. For activation, both compartments were squeezed together. The resulting mixture was applied by continuous rubbing on the enamel surface. Then, it was gently air-dried for 1 to 2 s. After that, the brackets were applied to the vestibular surface using the same resin and the same protocol as in the previous technique (Table 2).

All procedures were performed by the same operator. In the end, the samples were placed in distilled water to rehydrate and minimize the thermal effects due to the polymerization reaction. The samples were kept at 37° for 24 h.

**Figure 5:**
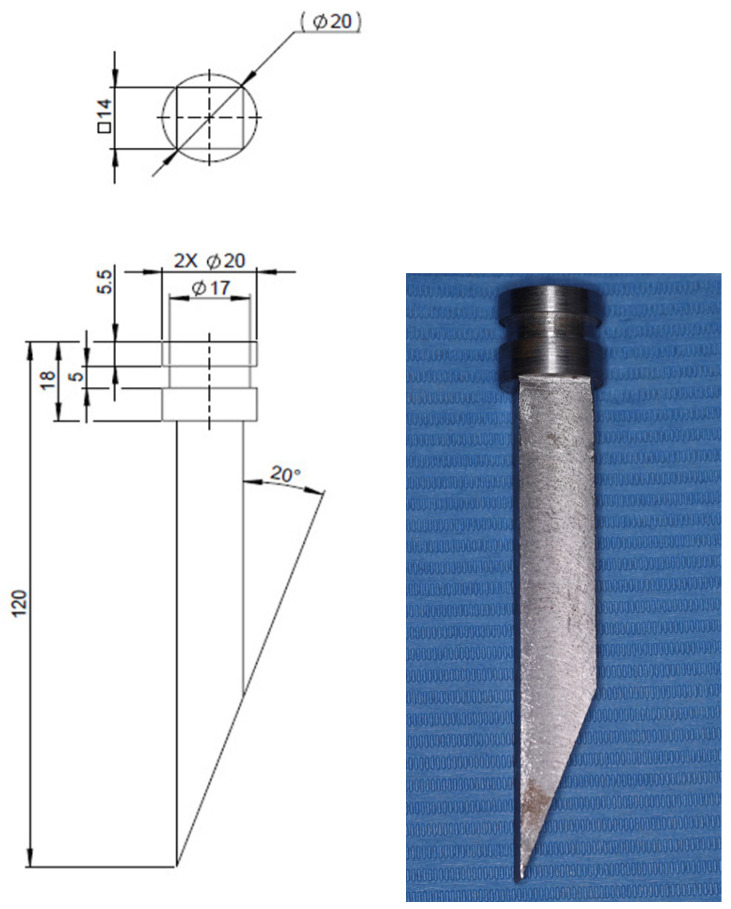
Sketch of the rod (knife) used to apply shear force to the adhesive at the bracket–tooth interface and the actual rod (knife).

After 24 h [41,52,53], the shear strength of the bracket–tooth adhesive bond was tested using a Laryee Universal Testing Machine (Laryee Technology Co., Beijing, China), equipped with a 10 kN force cell. Using an S235JRG1 steel rod (in accordance with STAS SR EN 10027-1:2017 [54]), an occlusal-gingival load was applied to the base of the bracket, thus producing a shear stress at the bracket–tooth interface. The rod (knife) was obtained by mechanical processing, starting from a Φ22 mm circular section semi-finished product made of S235JRG1 steel (according to STAS SR EN 10027-1:2017 [54], accessed on 7 July 2025). The monoblock, with a prismatic shape, ensures high rigidity and stability when transmitting forces. The shape and dimensions of the clamping head depend exclusively on the pin fastening device on the mobile crossbar of the testing machine. The cylindrical portion of the rod was obtained by roughing and finishing turning operations, ensuring the required concentricity and roughness for precise mounting in the clamping system of the testing machine. The prismatic portion was made by rough and finish milling, thus obtaining the specific geometry of the active edge. The active edge has a 20° cutting angle, designed to allow precise tangential placement on the enamel surface, directing the applied load strictly to the bracket–tooth interface. The 20° angle of the cutting edge was established based on geometric considerations, so that the tip of the rod has a lamellar profile with a linear active edge. With this approach, the applied force produces a predominantly shear stress at the bracket–tooth interface. This geometry allows controlled linear contact with the bracket base, minimizes the risk of local compression on the enamel, and ensures uniform transmission of the cutting force, increasing the reproducibility of the shear test. The shape and dimensions of the clamping head have been adapted to the pin-fixing system of the test machine’s movable crossbar, also ensuring the axial alignment of the applied force. The geometry adopted for the rod manufactured in this study complies with the principles defined in ISO 29022:2013 (Dentistry—Adhesion—Notched-edge shear bond strength test [55]), according to which the load must be applied parallel to the adhesion interface, ensuring a predominantly shear stress (Figure 5).

**Figure 6:**
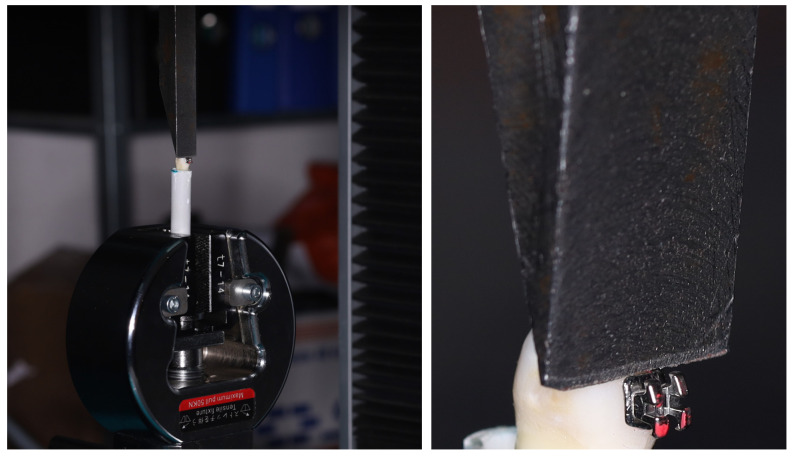
Shear bond strength testing.

The PVC tubes containing the embedded teeth were fixed so that the steel rod was parallel to the base of the bracket. The force was applied at a speed of 1 mm/min [28,41,56,57], until the bracket detached from the tooth surface. Using a computer (IBM Corp., Armonk, New York, USA) connected to the testing device, the shear force that caused bracket failure was recorded (Figure 6).

The variables collected in the study were:

Shear force, quantitative variable recorded in newtons;
Type of material used, nominal qualitative variable;
Type of bracket used, nominal qualitative variable;
Bracket base area, quantitative variable recorded in mm^2^.

The shear strength of the adhesive bond which is subjected to a shear force is usually expressed in megapascals (MPa) or N/mm^2^. It is denoted by τ_rf_ and is calculated according to the theory of Strength of Materials” as follows:

(1) $$\tau_{rf}=\frac{F}{A}\;\left[\frac{N}{{mm}^{2}}\right]$$

In Formula (1), F denotes the shear force and A denotes the area occupied by the adhesive, which corresponds to the base area of the bracket.

### 2.4. Ethical Considerations

The research was approved by the Academic Ethics and Deontology Committee of the University of Medicine and Pharmacy in Craiova (No. 253/6.11.2023). Patients gave their consent for the use of extracted teeth for orthodontic purposes by signing an informed consent form.

### 2.5. Statistical Analysis

The continuous measurements were reported as mean ± standard deviation (SD) and they were analyzed using Statistical Package for the Social Sciences (SPSS), version 26 (IBM Corp., Armonk, NY, USA). Their normality was analyzed using Shapiro–Wilk’s test. Subsequently, Levene’s test was employed for the equality of variances, and the Independent *t*-test was used for group comparisons, as well as the two-way ANOVA. For the present study, the value *p* < 0.05 was interpreted as statistically significant.

## 3. Results

The combination of the two bracket types and the two bonding systems resulted in four study groups.

*Shear bond strength analysis*

**Figure 7:**
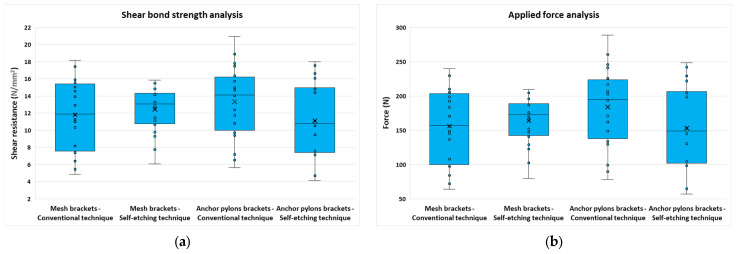
Bar graph showing the mean and standard deviation for the four groups: (**a**) shear bond strength; and (**b**) applied force.

The two types of brackets and the two bonding systems had similar shear strength (Figure 7). Slightly higher values were found in the group represented by the bonding of brackets with anchor pylons, using the conventional system (13.32 ± 4.20 N/mm^2^). Still, as Figure 7 indicates, the force and shear bond strength values follow very similar trends.

A two-way ANOVA was conducted to examine the effects of bonding systems and bracket type on the shear strength. Data are mean ± SD, unless otherwise stated. Residual analysis was performed to test for the assumptions of the two-way ANOVA. Outliers were assessed by inspection of a boxplot; normality was assessed using Shapiro–Wilk’s normality test for each cell of the design and homogeneity of variances was assessed by Levene’s test. There were no outliers, residuals were normally distributed (*p* > 0.05) and there was homogeneity of variances (*p* = 0.057).

The interaction effect between the bonding system and the type of bracket on the shear strength was not statistically significant, F(1, 76) = 2.582, *p* = 0.112, partial η^2^ = 0.033. Still, an analysis of the main effect for both parameters was performed, which indicated that the main effects were independently not statistically significant: F(1, 76) = 0.808, *p* = 0.371, partial η^2^ = 0.011 for the bonding system, and F(1, 76) = 0.011, *p* = 0.915, partial η^2^ < 0.0005 for the bracket type. The estimated marginal means of the shear strength for the conventional system and the self-etching system were 12.562 ± 0.627 and 11.764 ± 0.627, respectively. The estimated marginal means of the shear strength for mesh brackets and anchor pylons brackets were 12.115 ± 0.627 and 12.210 ± 0.627, respectively.

The ANOVA analysis did not reveal significant differences in shear strength or force depending on the type of bonding system used. The two types of brackets and the two types of bonding systems are similar in terms of shear strength and force values. There are no statistically significant interaction effects between them (Table 3).

*Applied force analysis*

Similarly, a two-way ANOVA was conducted to examine the effects of bonding system and bracket type on the force. There were no outliers and residuals were normally distributed (*p* > 0.05); however, there was no homogeneity of variances (*p* = 0.039). Since the group sample sizes are equal, there is normality and the ratio of the largest group variance to the smallest group variance is less than three, the two-way ANOVA is considered robust to heterogeneity of variance in these circumstances.

The interaction effect between the bonding system and the type of bracket on the force was not statistically significant, F(1, 76) = 2.622, *p* = 0.110, partial η^2^ = 0.033. Still, an analysis of the main effect for both parameters was performed, which indicated that the main effects were independently not statistically significant: F(1, 76) = 0.866, *p* = 0.355, partial η^2^ = 0.011 for the bonding system, and F(1, 76) = 0.507, *p* = 0.479, partial η^2^ = 0.007 for the bracket type. The estimated marginal means of the force for the conventional system and the self-etching system were 169.808 ± 8.511 and 158.609 ± 8.511, respectively. The estimated marginal means of the shear resistance for mesh brackets and anchor pylons brackets were 159.923 ± 8.511 and 168.494 ± 8.511, respectively.

Despite a non-statistically significant main effect of bonding system and type of bracket, the results from the ANOVA test reflect that the bonding system has a more pronounced effect upon the shear strength and the force, thus a subsequent analysis was performed upon the two parameters. A summary of the shear strength and force values for all groups is included in Table 4 and Table 5.

The above tables confirm that the force and shear strength values follow very similar trends. Differences between the bonding systems are more pronounced for anchor pylons brackets, but still not statistically significant.

## 4. Discussion

Dento-maxillary anomalies can cause various physical, psychological and functional disorders, affecting both oral and systemic health. A common consequence is the difficulty in maintaining oral hygiene, which predisposes individuals to dental caries, periodontal disease, and halitosis [58]. From a functional point of view, depending on the severity of the anomaly, affected individuals may experience chewing, phonation or temporomandibular joint disorders [59,60].

Moreover, the facial appearance is affected, which may lead to negative social interactions and the development of emotional and psychological problems. Oral health-related quality of life (OHQoL) is significantly compromised in a large proportion of affected individuals [61,62]. The high aesthetic standards imposed by contemporary society encourage patients to seek orthodontic treatment to achieve better social acceptance.

The success of orthodontic therapy depends on the effective, continuous application of forces to the teeth through the components of the orthodontic appliance. An essential element of treatment is the adhesion between the bracket and the enamel, as this ensures the stability of the bracket during treatment [63].

The efficacy of orthodontic adhesion is fundamentally governed by the physicochemical properties of the interface, specifically surface wettability and interface energy [64]. For successful resin infiltration, the surface energy of the enamel must significantly exceed the surface tension of the adhesive monomer. Phosphoric acid conditioning elevates this surface energy by removing the acquired pellicle and increasing the effective contact area through the creation of a porous architecture. This multiscale roughness facilitates micro- and meso-scale interlocking, where resin tags penetrate both the longitudinal enamel rod peripheries and the finer intra-rod structural voids, creating a spatially complex mechanical bond [4].

A particularly promising avenue in wet surface adhesion is inspired by the moisture-resistant properties of marine mussels. Mussels demonstrate extraordinary surface coating abilities in aquatic environments through catechol-based chemistry. For example, the utilization of a catechol-functionalized copolymer, poly(dopamine-methacrylate-co-2-methoxyethyl acrylate) (pDMA-MEA), as a primer for etch-and-rinse systems, has shown significant potential. Studies indicate that the architecture of pDMA-MEA is able to displace moisture and improve bond strength even in saliva-contaminated conditions, offering a solution to one of the most persistent challenges in clinical dentistry [65].

Recent advancements in material science have led to the development of Janus hydrogel, an innovative material that addresses the challenges of wet-tissue adhesion and the prevention of unwanted postoperative attachments. By precisely regulating the distribution of free hydroxyl and phenolic hydroxyl groups on the two opposite surfaces of the hydrogel, researchers have created a material with an asymmetric functional interface—a low-adhesion upper surface and a high-adhesion lower surface—which maintains its integrity across various wet tissues, even underwater.

Beyond their adhesive capacity, Janus hydrogels provide notable hemostatic properties and excellent biocompatibility as validated by cytocompatibility and hemolysis tests. This asymmetric functionality is particularly promising for intraoral applications, even in the field of adhesive dentistry or orthodontic bracket bonding.

The design and application of Janus hydrogels represent a highly interdisciplinary research frontier, integrating principles and methodologies from biology, medicine, physics, engineering, and computer science. Consequently, exploring the synergies between Janus hydrogels and emerging advanced technologies will be a critical trajectory for future investigations [66].

The bond between the enamel and the bracket can be analyzed at several levels as follows [26]: the bond between the tooth enamel and the adhesive, which is achieved through a chemical and physical mechanism; the bond between the adhesive and the bracket base (for metal brackets, in most cases, it is a purely mechanical connection [67]); intra-adhesive, referring to the properties of the adhesive material itself, its thickness and the photopolymerization method; and at the enamel level, in the context of a very strong bond, very high forces can cause enamel fracture during bracket removal.

Shear bond strength testing is the most used laboratory method for evaluating the strength of the adhesive bond between orthodontic brackets and tooth surface [68]. This is influenced by various factors, such as the type of primer, acid concentration, etching time, adhesive type, bracket design or clinical experience. Bracket detachment and rebonding can affect the enamel. Therefore, achieving an optimal adhesive bond is essential [37].

There are no clear guidelines in the literature regarding shear bond strength limits. An optimal orthodontic adhesive must create an adhesive bond that is strong enough to withstand masticatory forces, with a minimum strength of 5–10 MPa. On the other hand, it should not be too strong, to avoid fracturing the enamel when removing the bracket (40–50 MPa). Therefore, the ideal orthodontic adhesive should have a shear bond strength in the range of 5–50 MPa [37,69].

In the present study, the groups in which the conventional bonding systems were used (groups I and II) showed higher shear bond strength values. The average value was 12.562 ± 0.627 MPa, while for the groups in which the self-etching systems were used (groups III and IV), the average value was 11.764 ± 0.627 MPa.

Generally, studies evaluating shear bond strength in orthodontics show that the conventional bonding technique achieves higher shear bond strength compared to the self-etching technique, findings that are consistent with the results of the present study.

Given the high level of interest among specialists in this field, seven distinct studies published between 2014 and 2024 were reviewed. These studies evaluated the same adhesives as those used in the present study. The highest value for the conventional systems (34.75 ± 13.1 Mpa) was found in the study by Eren and Bilgiç [56]. The corresponding value for self-etching systems in the same study was 19 ± 7.3 MPa. The lowest reported shear bond strength for the conventional system was 9.38 ± 6.02 MPa [70], with its corresponding self-etching system result being 6.91 ± 3.58 MPa. Shalini et al. [41] reported 18.05 ± 4.2 MPa for the conventional system versus 16.2 ± 4 MPa for the self-etching system. Similarly, Zope et al. [71] reported 18.26 ± 7.50 MPa for the conventional system compared to 10.93 ± 4.02 MPa for the self-etching system. Sharma et al. [44] reported 15.49 ± 2.55 MPa for the conventional system, while reporting 11.57 ± 1.99 MPa for the self-etching system. The findings from Yadala et al. [72], which reported shear bond strength for conventional systems at 14.56 ± 2.91 MPa, closely align with the results obtained by Yillmaz et al. [51], which reported a value of 14.01 ± 5.79 MPa for the same systems. Regarding the self-etching systems, Yillmaz et al. [51] found a result of 10.13 ± 3.77 MPa, while Yadala et al. [72] reported 12.64 ± 2.56 MPa.

Despite this variability, the general conclusion drawn from studies is that conventional systems offer a greater shear bond strength for orthodontic bracket adhesion than the self-etching systems.

These findings, as part of in vitro studies, suggest that under strictly controlled, moisture-free conditions, conventional systems provide optimal retention. However, the clinical environment often presents challenges, particularly the contamination of dental surfaces with fluids such as water, blood, or saliva.

The data reported in other studies [73,74] provide a counterpoint to the in vitro results detailed above: under conditions of fluid contamination, self-etching systems may achieve superior shear bond strength (10.79 ± 2.43 MPa and 5.61 ± 1.02 MPa) compared to conventional systems (4.69 ± 3.10 MPa and 3.64 ± 1.28 MPa). Similar results have been reported by Sheikh et al., who attributed the findings to the hydrophilic nature of self-etching systems and their inherent acidity which helps them displace through macromolecules from the saliva and preserve the bond strength [75].

Moreover, on comparing saliva with blood, blood contamination produced the lowest shear bond strength. It could be because blood creates a greater mechanical barrier than saliva, given the difference in the type and amount of inorganic and organic elements in the blood [76]. As noted by Cunha et al. [77], in conditions of blood contamination self-etching systems maintained significantly higher shear bond strength values compared to the conventional hydrophobic system under contaminated conditions.

Therefore, self-etching systems may be a better option in certain clinical situations when optimal isolation cannot be achieved: surgically exposed impacted teeth, incompletely erupted teeth, and gingival bleeding. They can also be used in posterior areas, where isolation can be challenging, or in uncooperative patients [78].

The translation of in vitro data to the clinical environment requires a deep understanding of how fluid contamination interacts with the bracket’s mechanical retentive features.

For instance, while a 60- to 80-gauge foil mesh provides extensive surface area for micro-interlocking, these fine interstices may act as reservoirs for entrapped fluids, leading to localized interfacial nano-cracking and improper bracket attachment [79].

Conversely, laser-structured designs may exhibit superior moisture tolerance by facilitating a more continuous, uniform micro-interlocking adhesive pattern [80]. In this type of bracket base design, bond failure was reported at the resin–enamel interface due to maximum contact in the bracket base–resin interface [81].

The manufacturing process is also a critical determinant of bracket adhesion; brazed mesh bases are often superior to spot-welded designs because they eliminate “weld spots” that can obstruct adhesive flow and significantly reduce the effective surface area available for resin penetration. Moreover, they can serve as sites for stress concentration during debonding [82].

Furthermore, the biological substrate itself introduces significant variability. Unlike the sound premolar enamel typically utilized in laboratory studies, real-world clinical scenarios often involve hypomineralized enamel, fluorosis or irregular surface topographies. Shear bond strength values differ between fluorosed and non-fluorosed enamel surfaces when using conventional bonding protocols, with fluorosis reducing shear bond strength [83]. On the other hand, resin infiltration pretreatment (such as ICON) has been shown to enhance bracket adhesion and protect enamel integrity during debonding, especially in enamel with developmental defects or white spot lesions [84,85].

Orthodontic brackets bonding to hypomineralized enamel is influenced by surface preparation, with conventional bonding systems (especially when preceded by acidulated phosphate fluoride treatment) generally providing stronger bonds than self-etching systems [86].

Another objective of the study was to investigate the shear bond strength of two different types of brackets. Brackets with anchor pylons base design and 80-gauge mesh base design were selected. No significant differences were found, with the shear bond strength of the brackets with anchor pylons being 12.210 ± 0.627 MPa and that of the brackets with 80-gauge mesh being 12.115 ± 0.627 MPa. In the conventional technique, brackets with anchor pylons demonstrated greater shear bond strength (13.322 ± 4.202 MPa, compared to 11.802 ± 4.237 MPa), while in the self-etching technique, they reported a decrease in shear bond strength (11.099 ± 4.504 MPa, compared to 12.43 ± 2.663 MPa).

The two bonding systems have been extensively evaluated in the literature, but only the conventional system has been evaluated in combination with different bracket base designs. After searching for studies that used brackets with the same design as those evaluated in the present study, we identified only one study. Scribante et al. [87] reported the shear bond strength of 17.67 ± 6.90 MPa for brackets with anchor pylons and 13.78 ± 4.95 MPa for brackets with 80-gauge mesh base design.

The numerical trends observed in this study can be explained by the different dimensions and designs of the bracket bases. The anchor pylon base features a larger total surface area (13.8 mm^2^) compared to the surface of the 80-gauge mesh base (13.2 mm^2^). Unlike the traditional mesh, the pylon architecture provides a complex, three-dimensional geometry designed for deeper mechanical interlocking by increasing the resin penetration interface. However, it appears to be more sensitive to the enamel preparation method.

The conventional phosphoric acid etch creates a more aggressive retentive pattern, which likely allows the anchor pylon design to reach its full potential for a strong adhesive bond. In contrast, the milder self-etching primer resulted in lower numerical values for the pylon group, possibly due to insufficient etch depth for such a complex base. On the other hand, for the 80-gauge mesh brackets, the self-etching system yielded slightly higher values than the conventional protocol. This may indicate that the mesh grid is better suited for the thinner, more uniform hybrid layer created by self-etching primers. These results highlight the synergy between surface chemistry and base geometry.

The present study addresses shear bond strength by comparing two types of brackets with different base designs in combination with two types of adhesive systems. It also describes how the rod (knife) was designed. A review of the literature revealed that no other study provides detailed data regarding the manufacturing process of the rod (knife). The loading force was calculated to be precisely applied at the bracket–enamel interface. Biomechanically, applying a force (F) at a distance (L; e.g., bracket wings) from the base creates a moment (M = FxL), introducing tensile stresses at the upper margin of the bracket and compressive stresses at the lower margin. This complex loading pattern deviates from pure shear, often leading to premature bond failure at lower force values that do not accurately reflect the true interfacial adhesion. Our configuration optimizes reproducibility by ensuring that the rod makes contact as close as possible to the bracket–enamel interface. By minimizing the moment arm, the ‘peeling’ effect associated with rotational moments is reduced, thereby isolating the shear component of the bond. This approach is consistent with the recommendations of Eliades and Brantley [88].

The most important limitation of this study is its in vitro nature, which predominantly reflects a ‘dry’ environment and may not fully replicate the complex, fluctuating biological reality of the oral cavity. Also, patient-specific clinical variables such as gender, age, oral hygiene status, and dietary preferences were not recorded for the specimens used in this study. This is inherent to most in vitro shear bond strength research, where extracted teeth (typically collected for orthodontic reasons) are de-identified and provided without accompanying patient records. The focus is on the mechanical behavior of the bracket–adhesive–enamel interface rather than on patient characteristics. Methodological parameters (etching protocols, adhesive curing, storage conditions, thermocycling, crosshead speed, and force application) exert a more significant influence on shear bond strength outcomes than patient-specific clinical variables [89]. Nonetheless, it is acknowledged that clinical extrapolation must be made cautiously, and future in vivo and clinical studies incorporating patient level variables are desirable to confirm these findings under real oral conditions. The adhesive bond between the bracket and tooth surface is also influenced by other variables that are difficult to reproduce (variables strictly related to patient gender, age, oral hygiene, and dietary preferences, as described in our study previously. The present study provides short-term data; therefore, standardized long-term studies incorporating factors such as repeated masticatory loading and thermocycling to simulate temperature fluctuations, and prolonged exposure to saliva, are necessary to assess the shear bond strength of orthodontic brackets. Such investigations would provide more comprehensive data on the performance of different bracket base designs and adhesives in daily orthodontic practice. Moreover, future studies that include different enamel conditions (e.g., fluorosis and hypomineralization) or enamel surface contamination (e.g., saliva or blood) and long-term clinical follow-up are necessary to confirm the clinical applicability of the present findings and to understand how enamel variation and challenging bonding situations influence bracket performance.

## 5. Conclusions

The present study demonstrates that anchor pylon bracket bases achieve mechanical retention levels comparable to traditional 80-gauge mesh designs, with both architectures exhibiting resistance to shear forces. The data indicate that while the choice of adhesive system exerts influence on bonding performance, the results follow consistent trends across all experimental groups. Notably, the combination of anchor pylon brackets with a conventional bonding protocol yielded the highest mean shear bond strength. Regardless of the bracket–adhesive combination selected, the clinician can achieve bond strength values that assure the long-term stability of the bracket–tooth adhesion.

Further studies are necessary to expand the findings of the present study and to determine the influence of bracket base design and adhesive system on shear bond strength. Given that bracket bond failure is a common issue encountered in orthodontic practice, the analysis of shear bond strength should also be performed for rebonded brackets. The studies may also be conducted on molars or on various prosthetically restored surfaces. Additionally, it is essential that these studies be conducted under conditions of dental surface contamination.

## Figures and Tables

**Table 1:** Characteristics of each tested group.

Group	Coding	Number of Teeth	Material and Methods
Group I	I, respectively a number between 1 and 20	20	Conventional bonding system, metal brackets with 80-gauge mesh base
Group II	II, respectively a number between 1 and 20	20	Conventional bonding system, metal brackets with anchor pylons
Group III	III, respectively a number between 1 and 20	20	Self-etching bonding system, metal brackets with 80-gauge mesh base
Group IV	IV, respectively a number between 1 and 20	20	Self-etching bonding system, metal brackets with anchor pylons

**Table 2:** The materials used for each bonding technique [49,50,51].

Bonding Technique	Acid	Primer	Adhesive	Composition		
				Resin	Filler	Additional Contents
Conventional	Etching gel 37% (Reliance Orthodontic Products, Itasca, IL, USA)	Transbond XT Primer (3 M Unitek, St. Paul, MN, USA): Bis-GMA, TEGDMA, 4-(Dimethylamino)-Benzeneethanol	Transbond XT Light Cure Adhesive(3 M Unitek, St. Paul, MN, USA)	BisphenolA diglycidyl Ether dimethacrylate (10–20 wt%)Bisphenol A bis (2-hydroxyethyl ether) dimethacrylate (5– 10 wt%)	Silane-treated quartz (70–80 wt%)	Dichlorodimethylsilane reaction product with silica (<2 wt%)
Self-etching	-	Transbond™ Plus (3 M Unitek, St. Paul, MN, USA): 2-Propenoic acid, 2-methyl-, 2-hydroxyethyl ester, reaction products with phosphorus oxide (P2O5), DL-Camphorquinone, N,N-Dimethylbenzocaine, 4-Methoxyphenol, Hydroquinone	Transbond XT Light Cure Adhesive (3 M Unitek, St. Paul, MN, USA)	Bisphenol A diglycidyl ether dimethacrylate (10–20 wt%)Bisphenol A bis (2-hydroxyethyl ether) dimethacrylate (5– 10 wt%)	Silane-treated quartz (70–80 wt%)	Dichlorodimethylsilane reaction product with silica (<2 wt%)

**Table 3:** Results of the ANOVA factorial analysis for the bonding system and the type of bracket used.

Bracket	Shear Strength			Force		
	F(1, 76)	*p* *	Partial η^2^	F(1, 76)	*p* *	Partial η^2^
Bonding system	0.808	0.371	0.011	0.866	0.355	0.011
Bracket type	0.011	0.915	<0.0005	0.507	0.479	0.007
Interaction effect	2.582	0.112	0.033	2.622	0.110	0.033

* Two-way ANOVA.

**Table 4:** Shear resistance depending on bonding system and bracket type (mean ± SD).

Bracket	Conventional System	Self-Etching System	*p* *
Mesh brackets	11.802 ± 4.237	12.43 ± 2.663	0.579
Anchor pylons brackets	13.322 ± 4.202	11.099 ± 4.504	0.115
*p* *	0.262	0.264	

* Independent *t*-test.

**Table 5:** Applied force depending on bonding system and bracket type (mean ± SD).

Bracket	Conventional System	Self-Etching System	*p* *
Mesh brackets	155.777 ± 55.934	164.069 ± 35.149	0.578
Anchor pylons brackets	183.839 ± 57.984	153.149 ± 62.154	0.115
*p* *	0.128	0.499	

* Independent *t*-test.

## Data Availability

The data presented in this study are available on request from the corresponding author due to privacy, legal, and ethical restrictions.
